# Being the Family Caregiver of a Patient With Dementia During the Coronavirus Disease 2019 Lockdown

**DOI:** 10.3389/fnagi.2021.653533

**Published:** 2021-04-20

**Authors:** Milena Zucca, Valeria Isella, Raffaele Di Lorenzo, Camillo Marra, Annachiara Cagnin, Chiara Cupidi, Laura Bonanni, Valentina Laganà, Elisa Rubino, Nicola Vanacore, Federica Agosta, Paolo Caffarra, Renato Sambati, Davide Quaranta, Valeria Guglielmi, Ildebrando M. Appollonio, Giancarlo Logroscino, Massimo Filippi, Gioacchino Tedeschi, Carlo Ferrarese, Innocenzo Rainero, Amalia C. Bruni, the SINdem COVID-19 Study Group

**Affiliations:** ^1^Department of Neuroscience, Aging Brain and Memory Clinic, University of Torino, Turin, Italy; ^2^Department of Medicine and Surgery and Milan Center for Neuroscience (NeuroMi), University of Milano-Bicocca, Milan, Italy; ^3^Department of Primary Care, Regional Neurogenetic Centre, Catanzaro, Italy; ^4^Memory Clinic, Fondazione Policlinico Agostino Gemelli, IRCCS Università Cattolica del Sacro Cuore, Rome, Italy; ^5^Department of Neuroscience (DNS), University of Padua, Padua, Italy; ^6^CDCD Ospedale del Delta, AUSL Ferrara, Ferrara, Italy; ^7^Department of Neuroscience, Imaging and Clinical Sciences, University G. d’Annunzio of Chieti-Pescara, Chieti, Italy; ^8^Department of Neuroscience and Mental Health, AOU Città della Salute e della Scienza di Torino, Turin, Italy; ^9^National Institute of Health, Rome, Italy; ^10^Neurology Unit, Division of Neuroscience, IRCCS San Raffaele Scientific Institute, Milan, Italy; ^11^Neuroimaging Research Unit, Division of Neuroscience, IRCCS San Raffaele Scientific Institute, Milan, Italy; ^12^Vita-Salute San Raffaele University, Milan, Italy; ^13^Unit of Neuroscience, University of Parma, Parma, Italy; ^14^Department of Clinical Research in Neurology, Center for Neurodegenerative Diseases and the Aging Brain, University of Bari Aldo Moro, Bari, Italy; ^15^Department of Basic Medicine Neuroscience and Sense Organs, University of Bari Aldo Moro, Bari, Italy; ^16^Neurorehabilitation Unit, IRCCS San Raffaele Scientific Institute, Milan, Italy; ^17^Neurophysiology Service, IRCCS San Raffaele Scientific Institute, Milan, Italy; ^18^Department of Advanced Medical and Surgical Sciences, University of Campania “Luigi Vanvitelli”, Naples, Italy

**Keywords:** caregiver, dementia, COVID-19, stress, burden

## Abstract

**Background:** Family caregivers of patients with dementia are at high risk of stress and burden, and quarantine due to the coronavirus disease 2019 (COVID-19) pandemic may have increased the risk of psychological disturbances in this population. The current study was carried out during the national lockdown declared in March 2020 by the Italian government as a containment measure of the first wave of the coronavirus pandemic and is the first nationwide survey on the impact of COVID-19 lockdown on the mental health of dementia informal caregivers.

**Methods:** Eighty-seven dementia centers evenly distributed on the Italian territory enrolled 4,710 caregiver–patient pairs. Caregivers underwent a telephone interview assessing classical symptoms of caregiver stress and concern for the consequences of COVID-19 infection on patient’s health. We calculated prevalence of symptoms and regressed them on various potential stress risk factors: caregivers’ sociodemographic characteristics and lifestyle, patients’ clinical features, and lockdown-related elements, like discontinuity in medical care.

**Results:** Approximately 90% of caregivers reported at least one symptom of stress, and nearly 30% reported four or more symptoms. The most prevalent symptoms were concern for consequences of COVID-19 on patient’s health (75%) and anxiety (46%). The main risk factors for stress were identified as a conflicting relationship with the patient and discontinuity in assistance, but caregiver’s female sex, younger age, lower education, and cohabitation with the patient also had an impact. Availability of help from institutions or private individuals showed a protective effect against sense of abandonment but a detrimental effect on concern about the risk for the patient to contract COVID-19. The only protective factor was mild dementia severity, which was associated with a lower risk of feeling isolated and abandoned; type of dementia, on the other hand, did not affect stress risk.

**Conclusion:** Our results demonstrate the large prevalence of stress in family caregivers of patients with dementia during the COVID-19 pandemic and have identified both caregivers and situations at a higher risk of stress, which should be taken into account in the planning of interventions in support of quarantined families and patients.

## Introduction

Caregiver stress and burden, often described as an “enduring stress and frustration” phenomenon (Butcher et al., 2001), may have an extremely heavy impact on lives of family members who take care of relatives with dementia. Caregiver stress is mainly characterized by psychological symptoms such as anxiety, depression, irritability, feelings of being overwhelmed or abandonment, and tendency toward social isolation; but it is also associated with physical morbidity, disruption of family and professional life, and financial hardship (Faison et al., 1999; Chiao et al., 2015). Multiple factors have been shown to increase the risk of caregiver stress. Type of dementia, e.g., frontotemporal dementia (FTD) (Riedijk et al., 2006; Mioshi et al., 2013; D’Onofrio et al., 2015; Pilon et al., 2016; Liu et al., 2018), greater severity of cognitive and functional impairment (Wolfs et al., 2012; Mioshi et al., 2013; Liu et al., 2017; Riffin et al., 2019), and, most of all, worse behavioral disturbances (Wolfs et al., 2012; Papastavrou et al., 2007; Poon, 2019), have all been associated with higher levels of caregiver burden. Among carers’ characteristics, younger age, lower education, female gender, and some type of kinship, namely, being patient’s child, have also been linked with more severe anxiety and depression (Etters et al., 2008; Hughes et al., 2014). Finally, poor quality of the relationship between carer and care recipient (Faison et al., 1999; Steadman et al., 2007; Mioshi et al., 2013) and unavailability of social support and territorial resources (Upton and Reed, 2006) have also been shown to increase caregiver’s perceived burden.

In late 2019, a new infectious disease [coronavirus disease 2019 (COVID-19)] caused by severe acute respiratory syndrome coronavirus 2 (SARS-CoV-2) emerged in Wuhan, China, and subsequently spread in most countries and territories around the world. COVID-19 causes severe pneumonia and acute respiratory distress and may progress to multiple organ failure and death (Guan et al., 2020), especially in older adults and in patients with comorbidities (Shahid et al., 2020). In the absence of an antiviral treatment, anti-COVID interventions are now based on symptomatic therapy and on the prevention of contagion, which takes place through close contact with an infected person. In the majority of affected countries, national health institutions have therefore imposed periods of lockdown and mass quarantines, with extremely strict limitations on activities and movements, generally only allowed for work oremergencies. These measures have proved efficacious for the containment of the infection (Nussbaumer-Streit et al., 2020; Sebastiani et al., 2020), but requested restrictions are likely to have augmented the difficulties that family caregivers of patients with dementia have to deal with daily. Social isolation, limitation, and difficulties in accessing health and social support services, worsening of patient’s cognitive and motor deficits (Rainero et al., 2020), and behavioral and psychological symptoms (Cagnin et al., 2020) during the quarantine, in addition to dementia patients’ vulnerability to the viral infection, may all have increased the stress and burden perceived by dementia caregivers. Evidence in support of this hypothesis has in fact been provided by a few studies on pandemic-related caregiver stress performed in Italy (Carpinelli Mazzi et al., 2020; Altieri and Santangelo, 2021), the first epicenter of COVID-19 epidemic outside China, in two other European countries, Greece (Tsapanou et al., 2020) and Portugal (Borges-Machado et al., 2020), in Argentina (Cohen et al., 2020), and in India (Vaitheswaran et al., 2020). However, available data are scarce, and additional data are warranted. The current study was aimed at contributing to this literature and expanding knowledge on the impact of the coronavirus pandemic on caregiver stress. Published studies used telephone or online scales of caregiver burden, quality of life, or depression and anxiety and included a maximum of 239 participants. In our study, we relied on a nationwide survey carried out during the lockdown declared in Italy in the first COVID-19 wave, and we investigated an extensive array of stress symptoms and a comprehensive set of potential risk factors of higher caregiver stress due to the quarantine.

Northern Italy hosted the first European case of the coronavirus disease, in late February 2020; and Italy was the first country in the world to declare a national lockdown, on March 9, 2020. The Italian Neurological Society for Dementia (SINdem), a scientific society involved in dementia care and research, devised a nationwide survey on the effects of the quarantine on patients with dementia and their informal caregivers, which was carried out in April 2020 and involved nearly 5,000 caregiver–patient pairs. The survey was based on a telephone interview with family caregivers, which included a questionnaire on the worsening of patients’ motor, cognitive, and behavioral symptoms during the lockdown (Cagnin et al., 2020; Rainero et al., 2020), and a brief caregiver stress inventory. This paper reports on analysis of responses to the stress inventory, providing unique data on caregiver burden and related risk factors in such exceptional circumstances and in a particularly large cohort.

## Materials and Methods

Eighty-seven Italian Centers for Dementia and Cognitive Disorders (CDCD) were involved in the study and enrolled a total of 4,913 caregivers who participated on a voluntary basis. The only inclusion criterion was being the informal carer of a patient with dementia. Patients were included if they met criteria for one of the four most common forms of dementia (in regard to mixed dementia, patients were characterized according to predominant type of dementia): Alzheimer’s disease (AD), dementia with Lewy body disease (DLB), FTD, and vascular dementia (VaD). Moreover, for the present study, we only included caregivers of community-dwelling patients; hence, 203 caregivers of institutionalized patients who presented a different starting condition from other participants were excluded, leading to a final sample size of 4,710 cases. Their distribution on the Italian territory was homogeneous: 1,654 participants (35.0%) were from the north of Italy, 1,491 (32.0%) from the center of Italy, and 1,565 (33.0%) from the south. The total number of Italian regions involved was 16 out of 20.

### Caregiver Stress Questionnaire and Risk Profiling

Caregivers were contacted by telephone by a neurologist, a geriatrician, or a psychologist from patients’ referring CDCD and underwent a semi-structured interview after being informed about the study purpose and procedures and after giving oral consent to participate. All interviews were carried out from April 14 to April 24, 2020, i.e., from day 38 to day 48 from the start of the national lockdown (44.7 ± 1.2 days on average).

The survey protocol comprised a section reporting general information about the patient and the caregiver, an informant interview assessing changes in patient’s clinical conditions during the lockdown, and the caregiver stress questionnaire.

The caregiver stress questionnaire was composed of six binary present/absent questions tapping the following stress symptoms: (1) depression, (2) anxiety, (3) anguish, (4) irritability, (5) overwhelmed/helplessness (OH), and (6) isolation/abandonment (IA). In addition, a seventh question dealt with a caregiver’s concern for the consequences of COVID-19 infection on patient’s health. Caregivers were explicitly asked to respond focusing (1) on *changes* that occurred in their psychological status *since the beginning of the lockdown* and (2) on feelings related to *caregiving*, rather than to the pandemic or quarantine *per se*. Identification of risk factors for caregiver stress took into account caregivers’ and patients’ sociodemographic features, dementia characteristics, and factors related to the lockdown. Information about patient’s age and sex, disease stage, as defined by Clinical Dementia Rating (CDR) scale, and diagnosis of dementia were derived from CDCD’s clinical records, while the following data were collected during the phone interview: caregiver’s sex, age, and educational level; type of kinship (spouse, child, and other); cohabitation with the patient during the lockdown; presence of other family members at home; and temporary interruption of work activity (for professionally active caregivers). During the interview, the caregiver was also asked about presence of conflicts with the patient, availability of in-person help from other carers (relatives, friends, social services, or associations), and discontinuity in medical care during the quarantine.

### Ethical Standards

The study was initially approved by the Ethics Committee of the Coordinating Centre (University of Torino on April 7, 2020, no. 00150/2020) and then by the local ethics boards.

### Statistical Analysis

We conducted a descriptive analysis on the general characteristics of the study, prevalence of symptoms of caregiver stress, and frequency of risk factors. Mean, median, and standard deviation were produced for continuous variables, and frequencies and proportions for categorical variables. Rate of missing data was <2%; hence, no imputation was made. We performed logistic regression analyses in order to identify risk factors for caregiver stress. Each of the seven stress symptoms were first entered, as a dependent variable, in a series of *preliminary* uni- or multivariable regressions, with the following predictors or groups of predictors: caregiver’s and patient’s sex and age (<70 or ≥70 years), caregiver’s education (≤8 or >8 years of schooling), kinship (spouse, child, and other), cohabitation with the patient (yes/no), presence of other family members (yes/no), and interruption of work during lockdown (yes/no); type of dementia (AD/DLB/FTD/VaD) and disease stage (mild/moderate/severe or bedridden—the last two stages were pooled due to the low number of bedridden cases); presence of conflicts with the patient (present/absent); and availability of help from others (present/absent) and discontinuity in medical care or assistance for the patient (yes/no). Significant predictors were then entered in a *global* multivariable regression, one for each stress symptom as a dependent variable. Significance threshold was set at *p* < 0.05 for all analyses. All analyses were carried out with SPSS, version 26 (IBM Corp., 2019, Armonk, NY).

## Results

### General Characteristics of the Study Cohort

Study participants’ main features are shown in Table 1. The majority of caregivers were women (59.6%) and were patients’ children (53.6%). Their mean age was 59.5 ± 13.0 years, and mean education was 12.0 ± 4.3 years. In most cases (61.3%), caregivers lived with the patient and also with other family members (63.0%) and were not working during the quarantine (59.5%).

**TABLE 1:** General characteristics of the study cohort.

**Caregivers’ features**		

	**Mean**	**Standard deviation**
Age (years)	59.5	13.0
Education (years)	12.0	4.3
	***N*.**	**Valid%**
Sex (women)	2,809	59.6
**Kinship:**		
Spouse	1,731	37.3
Child	2,488	53.6
Others	425	9.2
Cohabitant with the patient (yes)	2,884	61.3
Other family members (yes)	2,958	63.0
Caregiver not working during lockdown	2,191/3,682*	59.5
Conflicts with the patient (yes)	1,072	22.8
Help from others (yes)	2,423	51.4
Discontinuity in care/assistance (yes)	1,094	23.4
*Number of professionally active caregivers.		

**Patients’ features**		

	**Mean**	**Standard deviation**

Age (years)	78.2	8.1
	**N.**	**Valid%**
Sex (women)	2,784	59.1
**Type of dementia:**		
Alzheimer’s disease	3,227	68.5
Dementia with Lewy bodies	339	7.2
Frontotemporal dementia	404	8.6
Vascular dementia	740	15.7
Disease stage by CDR: Mild	1,197	25.5
Moderate	2,317	49.5
Severe/bedridden	1,169	25.0

*CDR, Clinical Dementia Rating.*

Most patients had a diagnosis of AD (68.5%), and half were in a moderate disease stage (49.5%).

Only 22.8% of caregivers had experienced conflicts with the care-recipient.

Help from others was available for 51.4% of caregivers, while discontinuity in care and assistance during the lockdown was reported by 23.4%.

### Prevalence of Stress Symptoms

**FIGURE 1:**
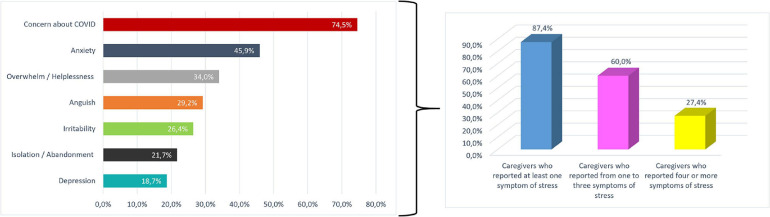
Overall prevalence of stress symptoms in the study cohort.

The vast majority of caregivers (4,116 subjects, 87.4%) reported at least one symptom of stress. Sixty percent (2,827 subjects) reported one to three symptoms, and 27.4% (1289 subjects) reported four or more symptoms. Concern about COVID-19 infection on health of patients with dementia was the most prevalent complaint, reported by 74.5% of participants, followed by anxiety and OH, reported, respectively, by 45.9 and 34.0% of participants; the other symptoms had a frequency ranging from 18.7 to 29.2% (Figure 1).

### Distribution of Stress Symptoms After Stratification of Caregiver Cohort by Various Characteristics of Interest

**FIGURE 2:**
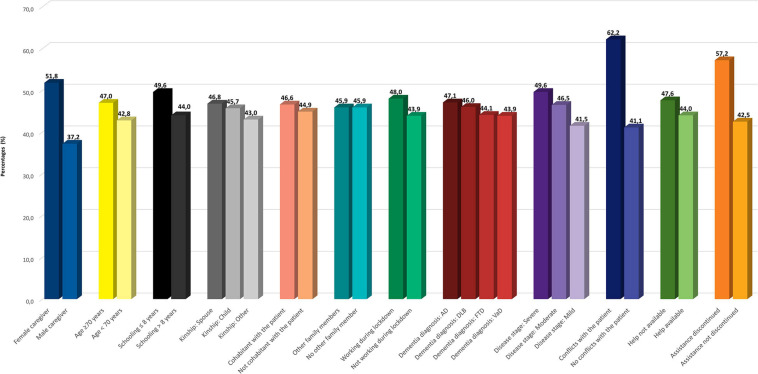
Prevalence of anxiety according to caregiver and patient features. Legend: AD, Alzheimer’s disease; FTD, frontotemporal dementia; DLB, dementia with Lewy bodies; VaD, vascular dementia.

**FIGURE 3:**
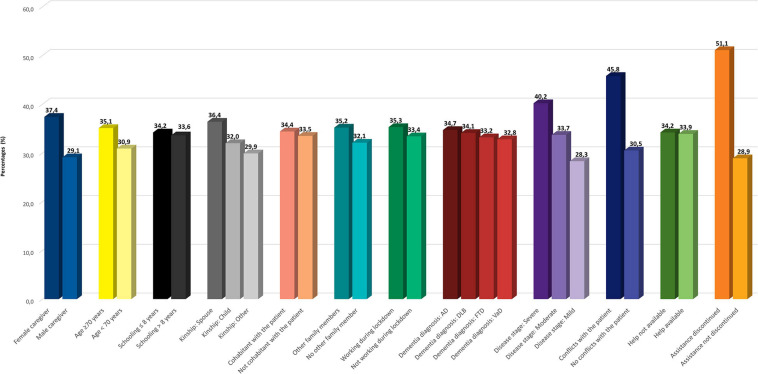
Prevalence of sense of being overwhelmed and helpless according to caregiver and patient features. Legend: AD, Alzheimer’s disease; FTD, frontotemporal dementia; DLB, dementia with Lewy bodies; VaD, vascular dementia.

A higher prevalence of all symptoms, and especially anxiety (Figure 2), OH (Figure 3), IA, and anguish (Supplementary Materials), was found for female caregivers, carers of patients with more severe dementia, and caregivers experiencing conflicts with the patient or discontinuity in medical assistance.

Anxiety (Figure 2) was more frequent also among carers who, due to lockdown restrictions, temporarily suspended work and who had contacts with people or institutions, which helped them in assisting the patient.

**FIGURE 4:**
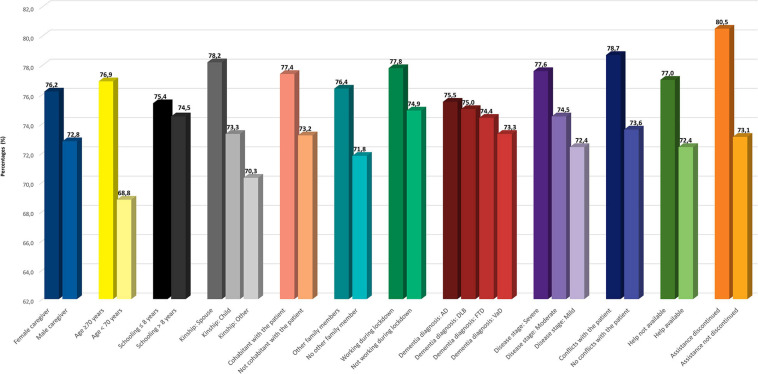
Prevalence of concern about consequences of COVID-19 on patient’s health according to caregiver and patient features. Legend: AD, Alzheimer’s disease; FTD, frontotemporal dementia; DLB, dementia with Lewy bodies; VaD, vascular dementia; COVID-19, coronavirus disease 2019.

Depression was found to be more prevalent in older, less educated caregivers and in spouses, than in other categories of relatives; in carers who lived with the care recipient, who did not live with other family members, and who had to interrupt work during lockdown; and in caregivers of patients with a diagnosis of FTD (Supplementary Materials). Only another stress symptom was affected by type of diagnosis: irritability was slightly more frequent in caregivers of patients with DLB (Supplementary Materials). Finally, concern about COVID (Figure 4) was more frequent in younger caregivers, in patient’s children, and in carers who were not cohabitant with the patient, and also in those who had contacts with helpers.

### Identification of Risk Factors of Stress Symptoms Through Regression Analyses

#### Results of Preliminary Univariate Regressions

Significant predictors of stress symptoms that emerged from preliminary logistic regressions are displayed in Table 2, while the Supplementary Table shows non-significant predictors (patient’s age and sex, presence of other family members at home, interruption of work activity, and type of dementia).

**TABLE 2:** Results (odds ratios and 95% confidence intervals) of preliminary regression analyses carried out for each stress symptom.

	Depression	Anxiety	Anguish	Irritability	Overwhelmed/helplessness	Isolation/abandonment	Concern for consequences of COVID on patient’s health
Female caregiver	1.59**** (1.32–1.90)	1.78**** (1.56–2.02)	1.85**** (1.59–2.14)	1.37**** (1.18–1.59)	1.47**** (1.28–1.68)	1.29** (1.10–1.52)	1.17* (1.01–1.36)
Caregiver’s age < 70 years	0.85 (0.64–1.14)	1.30* (1.02–1.64)	1.33* (1.02–1.72)	1.12 (0.86–1.47)	1.07 (0.83–1.37)	0.95 (0.71–1.25)	1.13 (0.87–1.46)
Caregiver’s education ≤ 8 years	1.27** (1.07–1.51)	1.27*** (1.11–1.46)	1.13 (0.98–1.32)	1.01 (0.86–1.17)	0.99 (0.86–1.15)	1.08 (0.92–1.27)	1.29** (1.11–1.52)
Kinship: child	1.49 (0.70–3.18)	1.86* (1.08–3.19)	1.39 (0.77–2.52)	2.05* (1.03–4.08)	1.74 (0.98–3.11)	1.23 (0.64–2.34)	1.63 (0.94–2.83)
Cohabitation with the patient	1.38** (1.13–1.69)	1.04 (0.90–1.22)	1.14 (0.97–1.35)	1.37*** (1.16–1.62)	1.17 (1.0–1.36)	1.40*** (1.16–1.67)	1.00 (0.84–1.20)
Dementia severity: mild	0.43 (0.19–1.01)	1.42 (0.63–3.18)	0.93 (0.39–2.22)	0.57 (0.25–1.29)	0.49 (0.23–1.07)	0.34** (0.15–0.76)	0.74 (0.30–1.86)
Conflicts with the patient	2.83**** (2.41–3.31)	2.36**** (2.05–2.72)	2.00**** (1.73–2.31)	2.78**** (2.40–3.21)	1.92**** (1.67–2.21)	2.96**** (2.54–3.44)	1.33*** (1.12–1.56)
Help from others	0.98 (0.84–1.13)	1.15* (1.02–1.29)	0.99 (0.87–1.13)	0.91 (0.80–1.04)	0.98 (0.86–1.10)	0.81* (0.70–0.93)	1.28*** (1.12–1.47)
Discontinuity in assistance	1.94**** (1.65–2.28)	1.81**** (1.58–2.07)	1.94**** (1.68–2.24)	2.34**** (2.03–2.71)	2.57**** (2.24–2.95)	3.58**** (3.08–4.16)	1.52**** (1.29–1.80)

Only predictors that were significant for at least one symptom are displayed. COVID, coronavirus disease. *p < 0.05, **p < 0.01, ***p < 0.001, ****p < 0.0001.

Female caregivers were more prone to develop stress symptoms of all types, but especially anxiety (OR 1.78) and anguish (OR 1.85); younger caregivers were more likely to show anxiety and anguish (ORs 1.30 and 1.33, respectively); and caregivers with a lower educational level tended to be at major risk of depression (OR 1.27), anxiety (OR 1.27), and concern about COVID infection (OR 1.29). Unlike other relatives, patients’ children were more likely to feel anxious (OR 1.86) and, above all, irritable (OR 2.03). Irritability was also more probable in caregivers who lived with the patient, together with depression and IA (with ORs ranging from 1.37 to 1.40).

Conflicts with the patient had a heavy negative impact on all stress symptoms. In particular, they caused a nearly threefold rise in the risk of IA (OR 2.96), depression (OR 2.83), and irritability (2.78).

Discontinuity in assistance was also strongly associated with a higher risk of stress, especially IA (OR 3.58), but also OH and irritability (ORs 2.57 and 2.34, respectively). On the other hand, availability of help increased anxiety (OR 1.15) and concern about COVID infection (OR 1.28), in spite of a protective effect against sense of abandonment (OR 0.81).

The only other protective factor was a mild dementia stage, which was associated with a minor risk of IA (OR 0.34).

#### Results of Multivariable Regressions

**FIGURE 5:**
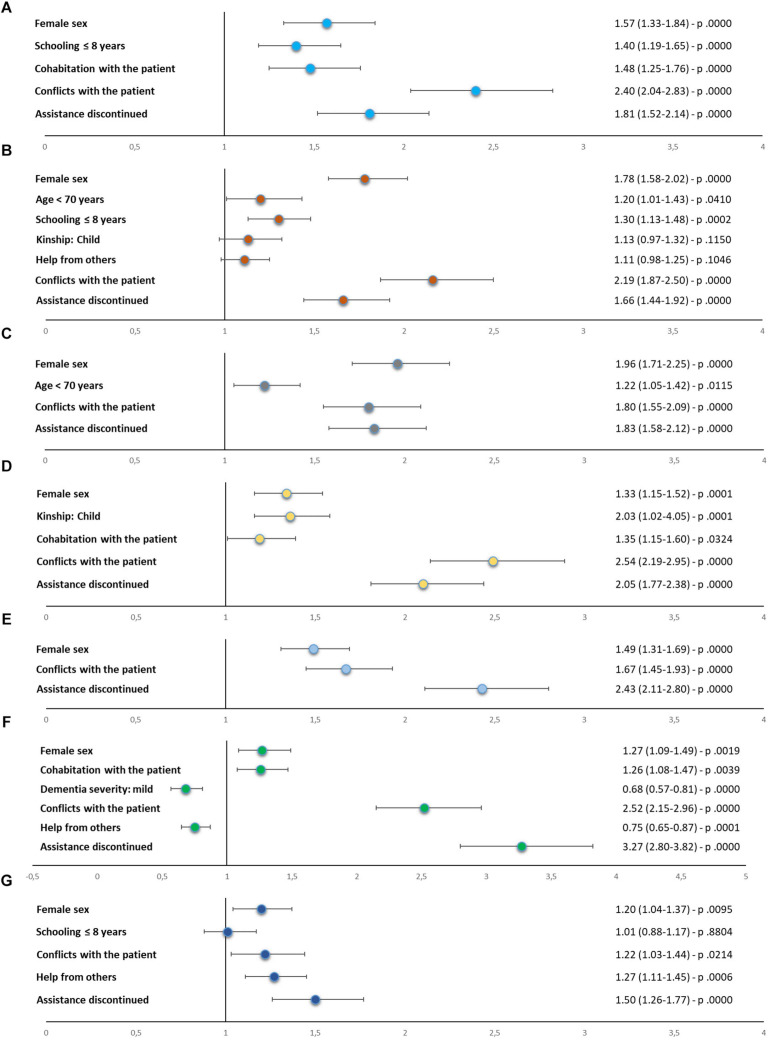
Results (odds ratios, 95% confidence intervals, and p values) of global regression analyses for identification of risk factors for each stress symptom: **(A)** depression, **(B)** anxiety, **(C)** anguish, **(D)** irritability, **(E)** sense of being overwhelmed/helplessness, **(F)** sense of isolation/abandonment, and **(G)** concern about consequences of COVID-19 on patient’s health. COVID-19, coronavirus disease 2019.

Almost all predictors that were significant at preliminary regressions were confirmed by global regressions (Figure 5). The only exceptions were the associations between caregivers’ age and education and concern about COVID, and type of kinship (child) and help from others and anxiety, which were no longer significant.

Female sex, presence of conflicts with the patient, and discontinuity in medical assistance were confirmed to increase the risk of all stress symptoms. In particular, female caregivers were more likely to feel anguished (OR 1.96) and anxious (OR 1.78); caregivers with a conflicting relationship with the patient were more likely to feel isolated/abandoned (OR 2.52), irritated (OR 2.49), and depressed (OR 2.40); and those experiencing discontinuation in assistance were more likely to develop sense of IA (OR 3.27) and OH (OR 2.43).

Global regressions also confirmed a higher risk of anxiety (OR 1.20) and anguish (OR 1.22) for younger caregivers, of anxiety (OR 1.30) and depression (OR 1.40) for caregivers with a lower educational level, and of irritability for patients’ children than for other relatives (OR 1.36).

Cohabitation with the patient was still a significant risk factor for depression (OR 1.48), irritability (OR 1.19), and IA (OR 1.26); and receiving help from others was confirmed to have a protective effect against IA (OR 0.75) but a detrimental effect on concern about COVID (OR 1.27).

Finally, carers of patients with mild dementia were confirmed to be at a lower risk of IA (OR 0.68).

## Discussion

To date, few studies have investigated the impact of the COVID-19 pandemic on the mental health of family caregivers of patients with dementia (Borges-Machado et al., 2020; Carpinelli Mazzi et al., 2020; Cohen et al., 2020; Tsapanou et al., 2020; Altieri and Santangelo, 2021), but this is the first nationwide multicenter survey, performed in Italy during the first wave of the coronavirus pandemic, that took into consideration a very large sample, a wide spectrum of symptoms of caregiver stress, and a comprehensive array of potential risk factors for higher stress levels.

Nearly 90% of our participants reported at least one symptom of stress, and nearly 20% reported four or more symptoms. In particular, anxiety and sense of being overwhelmed and helplessness were present in one in two and one in three caregivers, respectively, and were second only to concern about the consequences of COVID-19 infection on patient’s health, reported by three quarters of participants. Depression, anguish, irritability, and sense of isolation and abandonment were less common but were still the complaints of 20 to 30% of respondents.

Analysis of stress symptoms across subgroups of participants, stratified by various characteristics of interest (caregivers’ demographics, dementia features, and life conditions during the quarantine), and regression analyses assessing the relationship between these characteristics and stress symptoms outlined several risk factors for higher stress levels: caregiver’s female gender, younger age, and lower educational level, parent–child kinship, cohabitation with the care recipient, conflicts with the patient, and discontinuity in medical assistance due to lockdown were associated with a twofold or even threefold increase in the risk of developing symptoms within the anxiety or depression spectrum. Only two factors seemed to exert a protective effect against stress: carers of patients in a milder disease stage and those receiving help from institutions, associations, or individuals were at a lower risk of feeling isolated.

### Old Risk Factors for Caregiver Stress in a New Scenario

Most of the stressors identified by our survey are known determinants of caregiver stress in several prior studies (Pearlin et al., 1990; Faison et al., 1999; Rabinowitz et al., 2006; Campbell et al., 2008; Etters et al., 2008; Prince et al., 2012; Chiao et al., 2015; Liu et al., 2017; Carpinelli Mazzi et al., 2020). Cohabitation with the patient, conflicts between carer and care recipient, caregiver female sex, younger age, lower educational level, and close kinship tie with the patient have all been associated with increased caregiver perceived burden, depressed mood, feelings of isolation, anxiety, and also major physical and health problems (Faison et al., 1999; Campbell et al., 2008; Etters et al., 2008; Prince et al., 2012; Chiao et al., 2015).

In addition to confirming the association between stress and these well-known risk factors in our caregivers, we showed how a novel situation like the pandemic modulated such factors and their impact on caregiver burden. As an example, if we accept the use of schooling as a proxy measure for socioeconomic status (Hughes et al., 2014), we assume that less educated caregivers were more heavily affected by the economic consequences of the pandemic and that this contributed to raising their levels of perceived distress. Along the same line, carer–patient relationship was certainly hard-tested by the extremely strict limitations in movements, activities, and social contacts imposed by the quarantine, magnifying its impact on caregiver burden. A final example of how the quarantine modulated risk and protective factors of caregiver stress was the ambivalent reaction of our carers to availability of help from others. Our results confirmed the known effect of formal and informal social support in reducing burden of caregiving and feelings of isolation, as the risk of sense of abandonment was lower in the 50% of our participants who received support from acquaintances or services and associations. However, we also revealed the other side of the coin, since caregivers who took advantage of help during the lockdown were also more prone to be concerned about the risk, for their demented relative, to contract SARS-CoV-2 infection, probably through contact with helpers. Fear of spreading the disease while assisting patients had also been pointed out in another study on caregiver stress related to COVID-19 (Cohen et al., 2020) and will necessarily have to be taken into account in the planning of interventions in favor of quarantined patients and caregivers.

### The Pandemic and the Lockdown as a Novel Burden for the Caregiver

One of the strongest stressors that emerged from our survey, but also from similar studies (Carpinelli Mazzi et al., 2020; Cohen et al., 2020; Tsapanou et al., 2020), was discontinuation in medical care. This situation was reported only by 23% of our participants, probably due to the fact that the interview was performed a few weeks since the beginning of the lockdown, but its impact was quite heavy. Specifically, it put caregivers at a much higher risk of feeling isolated, abandoned, overwhelmed, and helpless. Interestingly, all these symptoms are typically associated with perception of an increased burden of care. As already suggested previously (Borges-Machado et al., 2020), caregivers in our cohort might have felt the responsibility to handle alone situations normally managed by, or in collaboration with, specialists or might have felt the load of having to find alternative ways to guarantee assistance to their loved ones.

The need for continuative medical care is known to be particularly intense in informal caregivers of dementia patients, and, when unmet, it is one of the main determinants of distress (Hughes et al., 2014). Importantly, specialist assistance has been shown to be relevant to caregivers not only for its medical content but also for its ability to boost caregivers’ confidence in their own competence and efficacy as carers, improving their mood, and also their capacity to deal with patients’ behavioral disturbances (Rabinowitz et al., 2006; Campbell et al., 2008).

### Pandemic-Related Stress: Confounder or Secondary Stressor?

Our caregiver stress questionnaire was aimed at detecting specifically carers’ psychological reactions to the strains of taking care of their relatives in a quarantine situation and was thus structured to induce responders to focus on changes in their mood and feelings, related to caregiving, rather than to the pandemic scenario itself. Nevertheless, this scenario has surely had an impact on responses to the questionnaire. Rather than being seen as a pure confounder, however, reactions of caregivers to the pandemic may be considered as a fundamental contributor of caregiver stress. One of the most influential and comprehensive models of stress process of dementia caregiving (Pearlin et al., 1990) distinguishes separate but highly interacting determinants of caregiver stress: “background/context” features, such as demographic, socioeconomic, and relational characteristics of the caregiver; “primary” stressors, anchored directly in caregiving, e.g., patient cognitive and functional deficits or behavioral disturbances; and “secondary” stressors, related to situations outside of the caregiver role. We suggest that the pandemic acted as a secondary stressor for our caregivers and that the influence of its psychological consequences (Luo et al., 2020) on responses to our interview added accuracy and completeness to the survey, rather than interfering with the data collection.

### The Role of Dementia Characteristics

In disagreement with past literature evidence, in our survey, we only found a minor impact of severity of dementia on stress levels and no impact of type of dementia. Patients with FTD or DLB present more severe neuropsychiatric symptoms than patients with AD, and those with AD present more severe cognitive deficits than those with VaD, and these clinical characteristics have been associated with higher burden for caregivers (Riedijk et al., 2006; Mioshi et al., 2013; D’Onofrio et al., 2015; Pilon et al., 2016; Liu et al., 2018). In our study, descriptive analysis showed a higher prevalence of depression in carers of patients with FTD and a slightly higher prevalence of irritability in carers of patients with DLB, but regression analysis did not identify dementia diagnosis as a significant, independent predictor. Within the framework of the model of Pearlin et al. (1990), this result may be seen as an interesting overturn in the relative impact of context and primary and secondary stressors on caregiver burden induced by the pandemic.

A possible account for this finding is suggested by the results of analyses of two subsets of data collected through the current survey and recently reported by Cagnin et al. (2020) and Rainero et al. (2020). These two studies investigated modifications of our patients’ cognitive, motor, and neuropsychiatric symptoms during the quarantine and reported a worsening in all forms of dementia, even if in different domains for different diagnoses (e.g., cognitive changes were major in AD and behavioral changes in DLB and FTD). The quarantine seems to have levelled out the differential impact of the various forms of dementia on caregiver burden.

### Study Limitations

Our study has some of the limitations of large multicenter studies. In particular, although items of caregiver stress questionnaire were straightforward, yes/no, questions, and interviewers followed a common procedure of assessment, there may have been variability in how questions were delivered and how responses were interpreted. Second, we did not use a standardized and validated scale for measuring caregiver stress. However, symptoms assessed in our interview are core symptoms included in the most used caregiver burden questionnaires (Zarit et al., 1980; Cohen et al., 1983; Hoefman et al., 2013). Third, all data were collected through a telephone interview because a face-to-face assessment was not possible due to the quarantine, and this may have increased the risks of misunderstandings, especially with older caregivers.

Finally, unlike other similar studies that were able to compare pre-lockdown and during-lockdown data (Borges-Machado et al., 2020; Altieri and Santangelo, 2021), we did not acquire information about caregivers’ mental state before the pandemic outbreak and the lockdown. Participants were asked expressly to focus on changes occurred during the quarantine, but their prior psychological conditions may have influenced their responses. This probably made our data less specific but added a naturalistic tenor, since caregiver stress related to the lockdown surely was the result of interaction of multiple, complex factors, including a caregiver’s baseline mood. Finally, even if we believe that our study cohort is representative of Italian family caregivers of patients with dementia, in virtue of the large sample size and of the homogeneous distribution of participating CDCD on the Italian territory, generalizability of our findings to other settings may be limited. Differences in caregivers’ sociodemographic characteristics and lifestyle, in organization of health and social systems, and also in the course of COVID-19 pandemic restrain applicability of our results to other populations. Also, our data cannot be generalized to caregivers of institutionalized patients, who were excluded from the current analyses.

### Implications for Interventions in Support of Caregivers

Despite the limitations discussed above, we believe that our survey has given a contribution to the knowledge of the consequences of the COVID-19 pandemic and quarantine on dementia caregivers and might have important psychosocial implications. First of all, we drew attention on the issue of the impact of the coronavirus disease on the mental health of informal carers of patients with dementia, and we provided a measure of the dimensions of this phenomenon with an exceptionally timely and large-scale study. Moreover, results of our risk profiling analysis identified a series of red flags that should be carefully scrutinized to detect situations and caregivers at greater risk of breakdown, hence in greater need of support. Finally, potentially useful indications have emerged for the planning of interventions targeted at the prevention of caregiver stress and relief of caregiver burden in quarantine situations. For instance, a conflicting patient–caregiver relationship might benefit from specific counseling, risks associated with contacts with support services might be contained through a reorganization of dispensation of social care, and interruptions in medical assistance might be overcome through potentiation of telemedicine. An increase of online services such as remote diagnosing and monitoring of patients, tele-consultation, online caregivers support, and patient tele-rehabilitation are potential promising solutions for counterbalancing the forced interruption imposed by the COVID-19 pandemic. Encouraging results were recently reported in reference to the efficacy of telehealth interventions to increase the psychological well-being of people with different types of dementia and their caregivers (Costanzo et al., 2020). However, although telemedicine can be a potential solution for the difficulties found in access to conventional health-care services, it is important to note that subjects with major neurocognitive disorders and/or with severe neurosensory deficits have greater difficulty in the management of online interventions especially if they are performed via audio-visual devices (Sekhon et al., 2021). In line with these suggestions, clinicians should consider adopting more often a combination of different and flexible telemedicine approaches to try and overcome these problems, making the use of telehealth services more effective and generalizable.

Such indications might even be transferred to “quarantine-like” scenarios unrelated with a pandemic, e.g., in cases of forced and prolonged cohabitation, problematic access to services, or restriction of social contacts.

## Data Availability Statement

The raw data supporting the conclusions of this article will be made available by the authors, without undue reservation.

## Ethics Statement

The studies involving human participants were reviewed and approved by Ethics Committee of University of Torino, Turin, Italy. Written informed consent for participation was not required for this study in accordance with the national legislation and the institutional requirements.

## Author Contributions

IR, AB, CM, AC, LB, CC, and VL designed the study and planned center recruitment. MZ and VI wrote the report. RDL did the statistical analyses. ER, VI, NV, FA, IA, PC, RS, DQ, VG, GL, MF, GT, and CF contributed to the interpretation and discussion of results and reviewed the manuscript. All authors contributed to the article and approved the submitted version.

## Conflict of Interest

The authors declare that the research was conducted in the absence of any commercial or financial relationships that could be construed as a potential conflict of interest.

## Appendix

### LIST OF COLLABORATING AUTHORS IN THE SINDEM COVID-19 STUDY GROUP:

**Aging Brain and Memory Clinic, Department of Neuroscience, University of Torino, Italy** (Erica Gallo, erica.gallo@unito.it; Alberto Grassini, alberto.grassini@unito.it; Andrea Marcinnò, andrea.marcinno@unito.it; Fausto Roveta, fausto.roveta@unito.it; Paola De Martino, paola.demartino@unito.it)

**Regional Neurogenetic Centre ASP Catanzaro, Italy** (Francesca Frangipane, francesca.frangipane@libero.it; Gianfranco Puccio, puccio@arn.it; Rosanna Colao, ros.colao@gmail.com; Maria Mirabelli, mariamirabelli@libero.it)

**Memory Clinic, Fondazione Policlinico Gemelli, Universit Cattolica del Sacro Cuore IRCCS, Rome, Italy** (Noemi Martellacci, noemi_18@hotmail.com; Federica Lino, federica.lino.psy@gmail.com)

**Department of Neuroscience, University of Padua, Italy** (Stefano Mozzetta, st.mozzetta@gmail.com; Cinzia Bussè, cinzia.busse@gmail.com)

**CDCD Aulss6 Alta Padovana, Italy** (Giulia Camporese, giulia.camporese@aulss6.veneto.it)

**Department of Biotechnological and Applied Clinical Sciences, Neurological Institute, University of L’Aquila, Italy** (Simona Sacco, simona.sacco@univaq.it)

**Avezzano Hospital, Avezzano, Italy** (Maria Carmela Lechiara, mlechiara@asl1abruzzo.it)

**Department of Neuroscience, Imaging and Clinical Sciences, University G. d’Annunzio, Chieti, Italy** (Claudia Carrarini, claudia.carrarini@live.it; Mirella Russo, mirella.russo92@gmail.com)

**Neurology Department, G. Mazzini Hospital, Teramo, Italy** (Alfonsina Casalena, alfonsinacasalena@yahoo.it)

**Clinica Neurologica San Salvatore Hospital, L’Aquila, Italy** (Patrizia Sucapane, p_sucapane@yahoo.com)

**Division of Neurology, Scientific Institute for Research, Hospitalization, and Care (IRCCS) Foundation “Carlo Besta” Neurological Institute, Milan, Italy** (Pietro Tiraboschi, Pietro.Tiraboschi@istituto-besta.it; Paola Caroppo, paola.caroppo@istituto-besta.it; Veronica Redaelli, veronica.redaelli@istitutobesta.it; Giuseppe Di Fede, giuseppe.difede@istituto-besta.it)

**CDCD Serra Spiga ASP Cosenza, Italy** (Daniela Coppa, dncoppa@gmail.com; Lenino Peluso, dott.pelusolenino@gmail.com)

**CDCD Polistena Laureana ASP Reggio Calabria, Cinquefrondi, Italy** (Pasqualina Insarda, linainsarda@tiscali.it)

**CDCD Jonio Sud District ASP Cosenza, Corigliano-Rossano, Italy** (Matteo De Bartolo, debartolo.matteo@libero.it)

**First Division of Neurology, University of Campania “Luigi Vanvitelli”, Naples, Italy** (Sabrina Esposito, sabrina.esposito1@unicampania.it)

**CDCD AORN “Ospedale dei Colli” – CTO, Naples, Italy** (Alessandro Iavarone, alessandro.iavarone@ospedalideicolli.it)

**CDCD DS 50, ASL Napoli 3 Sud, Naples, Italy** (Anna Vittoria Marta Orsini, annaorsini@virgilio.it)

**CDCD Neurologia, University of Campania “Federico II”, Naples, Italy** (Elena Salvatore, e.salvatore@unina.it; Chiara Criscuolo, sky569@hotmail.com)

**IRCCS Istituto delle Scienze Neurologiche di Bologna, UOC Clinica Neurologica Rete Neurologica Metropolitana (NEUROMET), Italy** (Luisa Sambati, luisasambati@gmail.com; Rossella Santoro, rossella.santoro@aosp.bo.it)

**AOU Sant’Anna di Icona – Ferrara, Italy** (Daniela Gragnaniello, d.gragnaniello@ospfe.it)

**CDCD Ospedale del Delta, AUSL Ferrara, Italy** (Ilaria Pedriali, ilaria.pedriali@ospfe.it)

**CDCD AUSL Parma, Italy** (Livia Ludovico, lludovico@ausl.pr.it)

**AUO Policlinico Modena, Italy** (Annalisa Chiari, chiari.annalisa@aou.mo.it)

**UOC Cognitive Disorders and Dementia, Department of Primary Care, AUSL Modena, Italy** (Andrea Fabbo, a.fabbo@ausl.mo.it; Petra Bevilacqua, p.bevilacqua@ausl.mo.it; Chiara Galli, ch.galli@ausl.mo.it; Silvia Magarelli, s.magarelli@ausl.mo.it)

**A.I.M.A. sez Parma, Italy** (Marta Perini, perini_marta@libero.it)

**Fondazione Santa Lucia IRCCS, Rome, Italy & Menninger Department of Psychiatry and Behavioural Sciences, Baylor College of Medicine, Houston, Tx, USA** (Gianfranco Spalletta, g.spalletta@hsantalucia.it)

**Fondazione Santa Lucia IRCCS, Rome, Italy** (Nerisa Banaj, n.banaj@hsantalucia.it; Desirée Estela Porcari, de.porcari@hsantalucia.it; Giulia Caruso, g.caruso@hsantalucia.it)

**Dipartimento di Neuroscienze, Universit di Roma “Tor Vergata”, Rome, Italy** (Desirée Estela Porcari)

**AOU Sant’Andrea, Rome, Italy** (Virginia Cipollini, virginiacipollini@uniroma1.it)

**AO San Giovanni Addolorata, Rome, Italy** (Anna Rosa Casini, arosa.casini@virgilio.it)

**Campus Biomedico, University of Roma, Italy** (Francesca Ursini, f.ursini@unicampus.it)

**Department of Neuroscience, University of Roma “La Sapienza”, Italy** (Giuseppe Bruno, giuseppe.bruno@uniroma1.it)

**Department of Geriatrics Fondazione Poliambulanza di Brescia, Italy** (Renzo Rozzini, renzo.rozzini@poliambulanza.it)

**Luigi Sacco Hospital, University of Milano, Italy** (Michela Brambilla, michela.brambilla@libero.it)

**Unit of Neurology, IRCCS San Raffaele Scientific Institute and Vita-Salute San Raffaele University, Milan, Italy** (Giuseppe Magnani, magnani.giuseppe@hsr.it; Francesca Caso, caso.francesca@hsr.it; Edoardo G. Spinelli, spinelli.edoardogioele@hsr.it)

**Unit of Behavioral Neurology IRCCS Mondino Foundation, and Department of Brain and Behavioral Sciences, University of Pavia, Italy** (Matteo Cotta Ramusino, matteo.cottaramusino@mondino.it; Giulia Perini, giulia.perini@mondino.it)

**Department of Experimental and Clinical Medicine, Polytechnic University of Marche, Ancona, Italy** (Simona Luzzi, s.luzzi@staff.univpm.it)

**CDCD Mazzoni Hospital, Ascoli Piceno, Italy** (Gabriella Cacchiò, cacchiogabriella@tiscali.it)

**CDCD Area Vasta 4, Fermo, Italy** (Alessia Ciccola, cdcdneurologia.av4@sanita.marche.it; Lorena Cionfrini, cdcdneurologia@sanita.marche.it)

**Geriatric Operative Unit, IRCCS-INRCA, Fermo, Italy** (Cinzia Giuli, c.giuli@inrca.it)

**CDCD Area Vasta 3, Macerata, Italy** (Katia Fabi, katia.fabi@libero.it)

**Azienda Ospedaliera Marche Nord, Pesaro, Italy** (Marco Guidi, marcoguidi55@gmail.com)

**CDCD San Benedetto del Tronto, Italy** (Cristina Paci, cpaci@libero.it)

**CDCD IRCSS Neuromed di Pozzilli, Isernia, Italy** (Annaelisa Castellano, annaelisacastellano@yahoo.it)

**Neurodegenerative Centre, University of Bari “Aldo Moro”, Bari, Italy** (Rossella Petrucci, rpetrucci78@gmail.com; Miriam Accogli)

**Neurology Department, Foggia University Hospital, Foggia, Italy** (Elena Carapelle, elecarpi@hotmail.it)

**CDCD of Casarano, Lecce, Italy** (Gianluigi Calabrese, gianluigicalabrese@libero.it)

**CDCD DSS of Campi Salentina, Lecce, Italy** (Giovanna Nicoletta Trevisi, trevisigiovanna@libero.it)

**CDCD DSS of Lecce, Italy** (Brigida Coluccia, colbrig@libero.it)

**CDCD DSS of Maglie, Italy** (Antonella Vasquez Giuliano)

**CDCD Ospedale Vito Fazzi Lecce, Italy** (Marcella Caggiula)

**Department of Neurology, University of Milano – Bicocca, Italy** (Valentina Impagnatiello, valentina.impagnatiello@gmail.com; Francesca Beretta, f.beretta30@campus.unimib.it)

**CDCD PO Santissima Trinit , ASSL Cagliari, Italy** (Antonio Milia, antoniomilia55@gmail.com; Giuseppina Pilia, giusi.pilia77@gmail.com; Maria Giuseppina Mascia, mgmascia@gmail.com)

**CDCD Area Vasta 1, Cagliari, Italy** (Valeria Putzu, putzu.valeria@tiscali.it)

**Section of Neurology Department of Biomedicine, Neuroscience and Advanced Diagnostics, University of Palermo, Italy** (Tommaso Piccoli, tommaso.piccoli@gmail.com; Luca Cuffaro, cuffaro.luca@gmail.com; Roberto Monastero, roberto.monastero@gmail.com)

**Neurology and Neurophysiopathology Unit, AOUP “Paolo Giaccone”, Palermo, Italy** (Antonella Battaglia, antobatt1994@yahoo.it; Valeria Blandino, valeriabl@libero.it; Federica Lupo, federicalupo1@gmail.com)

**UO Neurodegenerative Disorders, ASP 2, Caltanissetta, Italy** (Eduardo Cumbo, eduardo.cumbo@tiscali.it)

**AOU Policlinico “Vittorio Emanuele”, Catania, Italy** (Antonina Luca, antolucaster@gmail.com)

**AO Cannizzaro, Catania, Italy** (Giuseppe Caravaglios, giuseppe.caravaglios@gmail.com)

**Psychogeriatric Unit, ASP Messina, Italy** (Annalisa Vezzosi, psicogeriatria@asp.messina.it)

**Neurology I, Department of Neuroscience, Psychology, Drug Research and Child Health, AOU Careggi, Florence, Italy** (Valentina Bessi, valentina.bessi@unifi.it)

**CDCD, Neurology I, AOU University of Pisa, Italy** (Gloria Tognoni, gloria.tognoni@med.unipi.it)

**Geriatric Unit, Department of Clinical and Experimental Medicine, University of Pisa, Italy** (Valeria Calsolaro, valina82@gmail.com)

**CDCD, Division of Geriatric and Intensive Care Medicine, AOU Careggi and University of Florence, Italy** (Enrico Mossello)

**CDCD Territoriale, USL Umbria 1, Perugia, Italy** (Serena Amici, serena.amici@uslumbrial.it; Alberto Trequattrini, alberto.trequattrini@uslumbria1.it; Salvatore Pezzuto, salvatore.pezzuto@uslumbria1.it)

**Department of Medicine, University of Perugia, Italy** (Patrizia Mecocci, patrizia.mecocci@unipg.it, Giulia Fichera, giuliafcr@gmail.com)

**CDCD AUSSL 7 Pedemontana, Bassano del Grappa, Italy** (Samantha Pradelli, samantha.pradelli@aulss7.veneto.it)

**CDCD Geriatria, Dolo, Venice, Italy** (Marino Formilan, uva.geriatriadolo@aulss3.veneto.it)

**CDCD Geriatric Unit, University of Padua, Italy** (Alessandra Coin, alessandra.coin@unipd.it)

**CDCD AULSS 9 Scaligera, Verona, Italy** (Laura De Togni, laura.detogni@auiss9.veneto.it; Francesca Sala, francesca.sala@aulss9.veneto.it; Valentina Nicolosi, neuropsicologia.villafranca@aulss9.veneto.it)

**CDCD AULSS 2 Marca Trevigiana, Treviso, Italy** (Maurizio Gallucci, maurizio.gallucci@aulss2.veneto.it; Anna Paola Mazzarolo, annapaola.mazzarolo@aulss2.veneto.it; Cristina Bergamelli, cristina.bergamelli@aulss2.veneto.it)
