# Supplementary material for: Being the Family Caregiver of a Patient With Dementia During the Coronavirus Disease 2019 Lockdown
Source: Front Aging Neurosci. 2021 Apr 20;13:653533. doi: 10.3389/fnagi.2021.653533 (PMC8098661; doi:10.3389/fnagi.2021.653533)
Supplement: Supplementary file 1 [file Table_1.DOCX]

| SUPPLEMENTARY TABLE.  Odds ratios and 95% confidence intervals of independent variables included in preliminary regression analyses but excluded from the model as not significant. | | | | | | | |
| --- | --- | --- | --- | --- | --- | --- | --- |
|  | **Depression** | **Anxiety** | **Anguish** | **Irritability** | **Overwhelm / Helplessness** | **Isolation / Abandonment** | **Concern for consequences of COVID on patient’s health** |
| KINSHIP: |  |  |  |  |  |  |  |
| Spouse | 1.53  (0.70 – 3.34) | 1.66  (0.94 – 2.91) | 1.31  (0.70 – 2.44) | 1.63  (0.80 – 3.32) | 1.27  (0.69 – 2.32) | 0.98  (0.50 – 1.91) | 1.04  (0.58 – 1.85) |
| Other | 1.33  (0.60 – 2.95) | 1.52  (0.86 – 2.68) | 1.20  (0.64 – 2.25) | 1.83  (0.89 – 3.75) | 1.26  (0.68 – 2.32) | 1.15  (0.58 – 2.26) | 1.25  (0.70 – 2.25) |
| Presence of other  family members | 0.99  (0.83 – 1.18) | 0.96  (0.83 – 1.11) | 1.09  (0.93 – 1.28) | 1.02  (0.87 – 1.19) | 1.07  (0.92 – 1.25) | 1.03  (0.87 – 1.22) | 1.04  (0.89 – 1.22) |
|  |  |  |  |  |  |  |  |
| Working during lockdown | 0.85  (0.62 – 1.16) | 0.85  (0.67 – 1.10) | 0.81  (0.61 – 1.07) | 1.06  (0.80 – 1.42) | 0.86  (0.66 – 1.12) | 1.01  (0.75 – 1.37) | 1.12  (0.85 – 1.48) |
|  |  |  |  |  |  |  |  |
| Female patient | 0.99  (0.82 – 1.19) | 0.99  (0.86 – 1.14) | 1.01  (0.87 – 1.18) | 0.92  (0.78 – 1.07) | 1.00  (0.86 – 1.15) | 0.99  (0.84 – 1.17) | 1.13  (0.96 – 1.32) |
| Patient's age < 70 years | 1.0  (0.99 – 1.01) | 1.0  (0.99 – 1.01) | 1.01  (0.98 – 1.02) | 1.00  (0.99 – 1.01) | 1.0  (0.99 – 1.01) | 0.99  (0.98 – 1.00) | 0.99  (0.98 – 1.00) |
| SEVERITY OF DEMENTIA: |  |  |  |  |  |  |  |
| Moderate | 0.53  (0.23 – 1.23) | 1.74  (0.78 – 3.89) | 1.18  (0.50 – 2.81) | 0.75  (0.34 – 1.69) | 0.64  (0.30 – 1.37) | 0.47  (0.21 – 1.03) | 0.83  (0.33 – 2.06) |
| Severe | 0.78  (0.33 – 1.78) | 1.97  (0.88 – 4.43) | 1.59  (0.67 – 3.80) | 0.84  (0.37 – 1.88) | 0.84  (0.39 – 1.82) | 0.66  (0.30 – 1.45) | 0.99  (0.39 – 2.47) |
| TYPE OF DEMENTIA: |  |  |  |  |  |  |  |
| Alzheimer's Disease | 0.53  (0.00 - >1.0e12) | 0.77  (0.00 - >1.0e12) | 0.73  (0.00 - >1.0e12) | 0.66  (0.00 - >1.0e12) | 2.23  (0.00 - >1.0e12) | 0.98  (0.00 - >1.0e12) | 0.75  (0.00 - >1.0e12) |
| Dementia with Lewy bodies | 0.66  (0.00 - >1.0e12) | 0.70  (0.00 - >1.0e12) | 0.78  (0.00 - >1.0e12) | 0.85  (0.00 - >1.0e12) | 2.11  (0.00 - >1.0e12) | 1.04  (0.00 - >1.0e12) | 0.77  (0.00 - >1.0e12) |
| Frontotemporal Dementia | 0.83  (0.00 - >1.0e12) | 0.71  (0.00 - >1.0e12) | 0.88  (0.00 - >1.0e12) | 0.79  (0.00 - >1.0e12) | 2.12  (0.00 - >1.0e12) | 1.18  (0.00 - >1.0e12) | 0.68  (0.00 - >1.0e12) |
| Vascular Dementia | 0.67  (0.00 - >1.0e12) | 0.81  (0.00 - >1.0e12) | 0.88  (0.00 - >1.0e12) | 0.79  (0.00 - >1.0e12) | 2.31  (0.00 - >1.0e12) | 1.11  (0.00 - >1.0e12) | 0.73  (0.00 - >1.0e12) |

Prevalence of sense of Isolation and Abandonment according to caregiver and patient features.


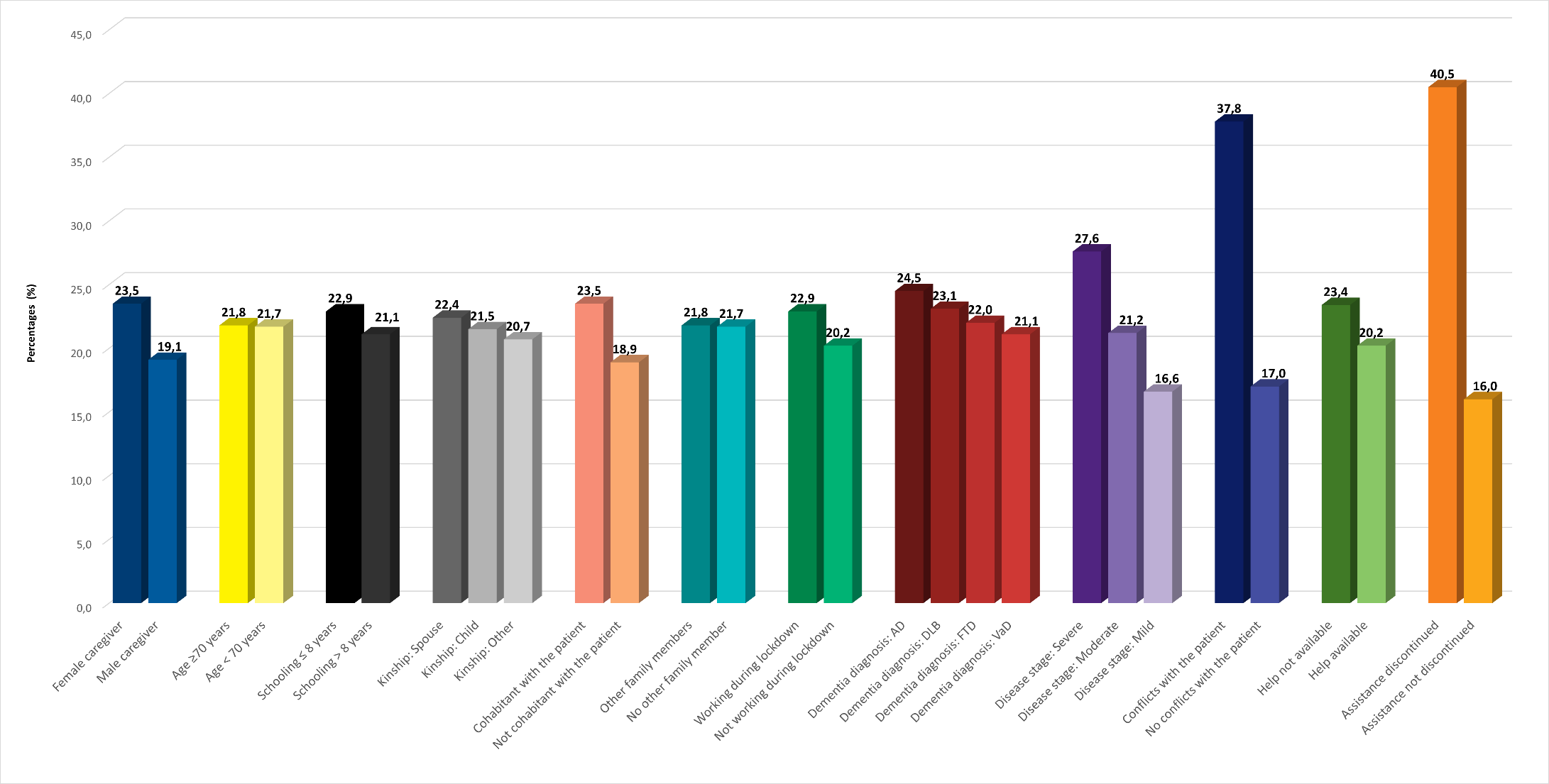


Legend: AD= Alzheimer's Disease, FTD= Frontotemporal Dementia, DLB= Dementia with Lewy bodies, VaD= Vascular Dementia

Prevalence of Anguish according to caregiver and patient features.


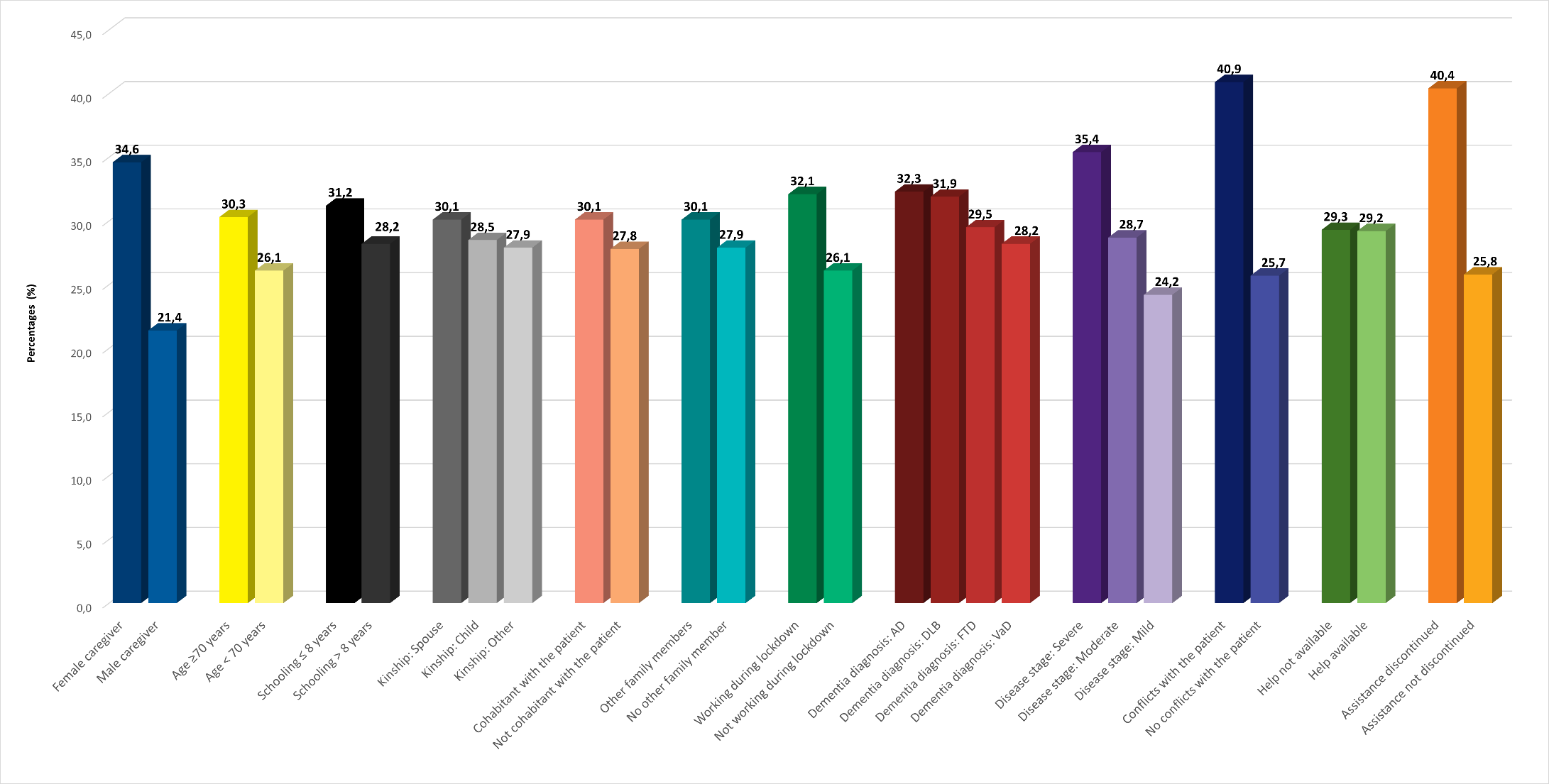


Legend: AD= Alzheimer's Disease, FTD= Frontotemporal Dementia, DLB= Dementia with Lewy bodies, VaD= Vascular Dementia

Prevalence of Depression according to caregiver and patient features.


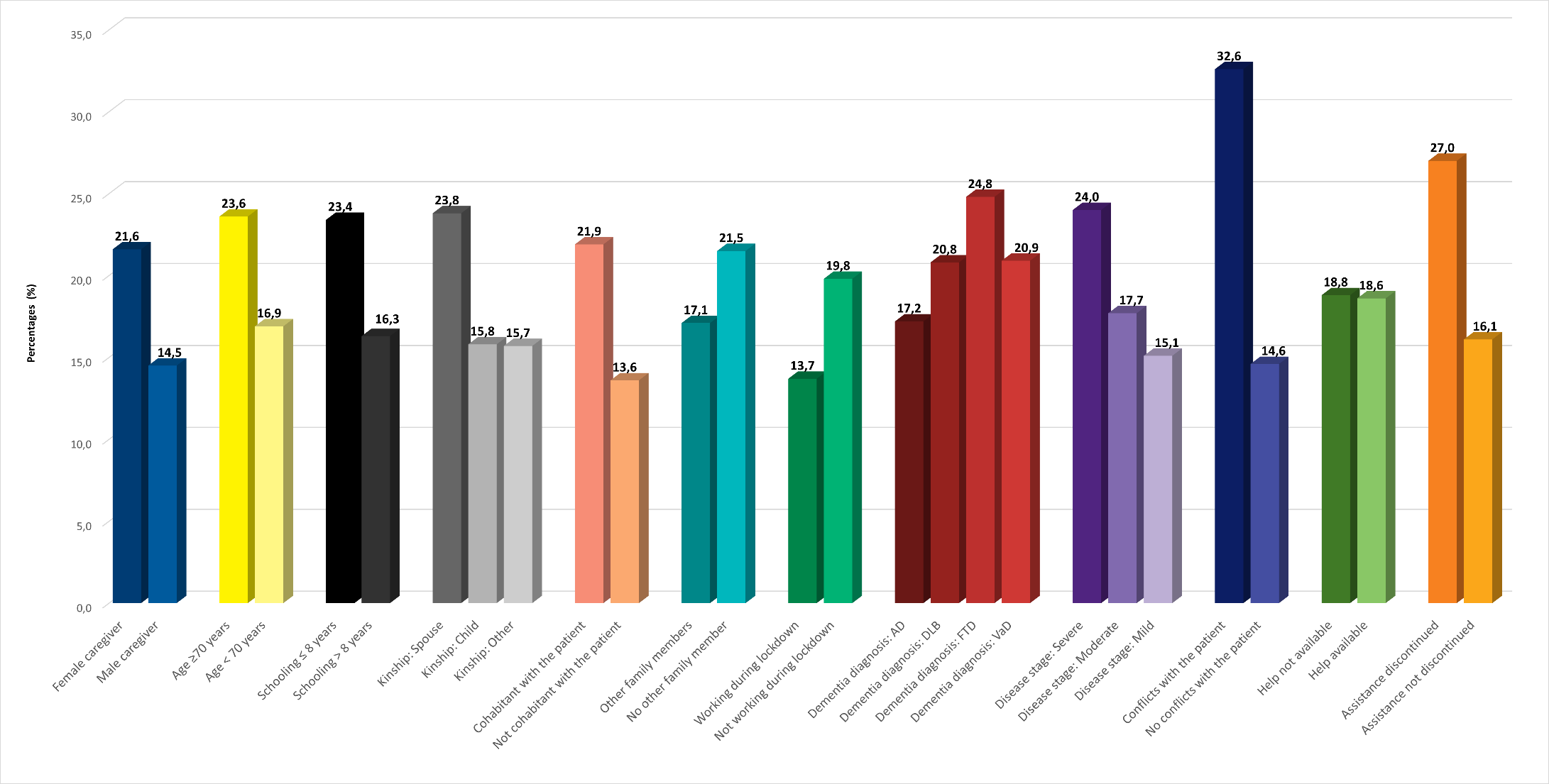


Legend: AD= Alzheimer's Disease, FTD= Frontotemporal Dementia, DLB= Dementia with Lewy bodies, VaD= Vascular Dementia

Prevalence of Irritability according to caregiver and patient features.


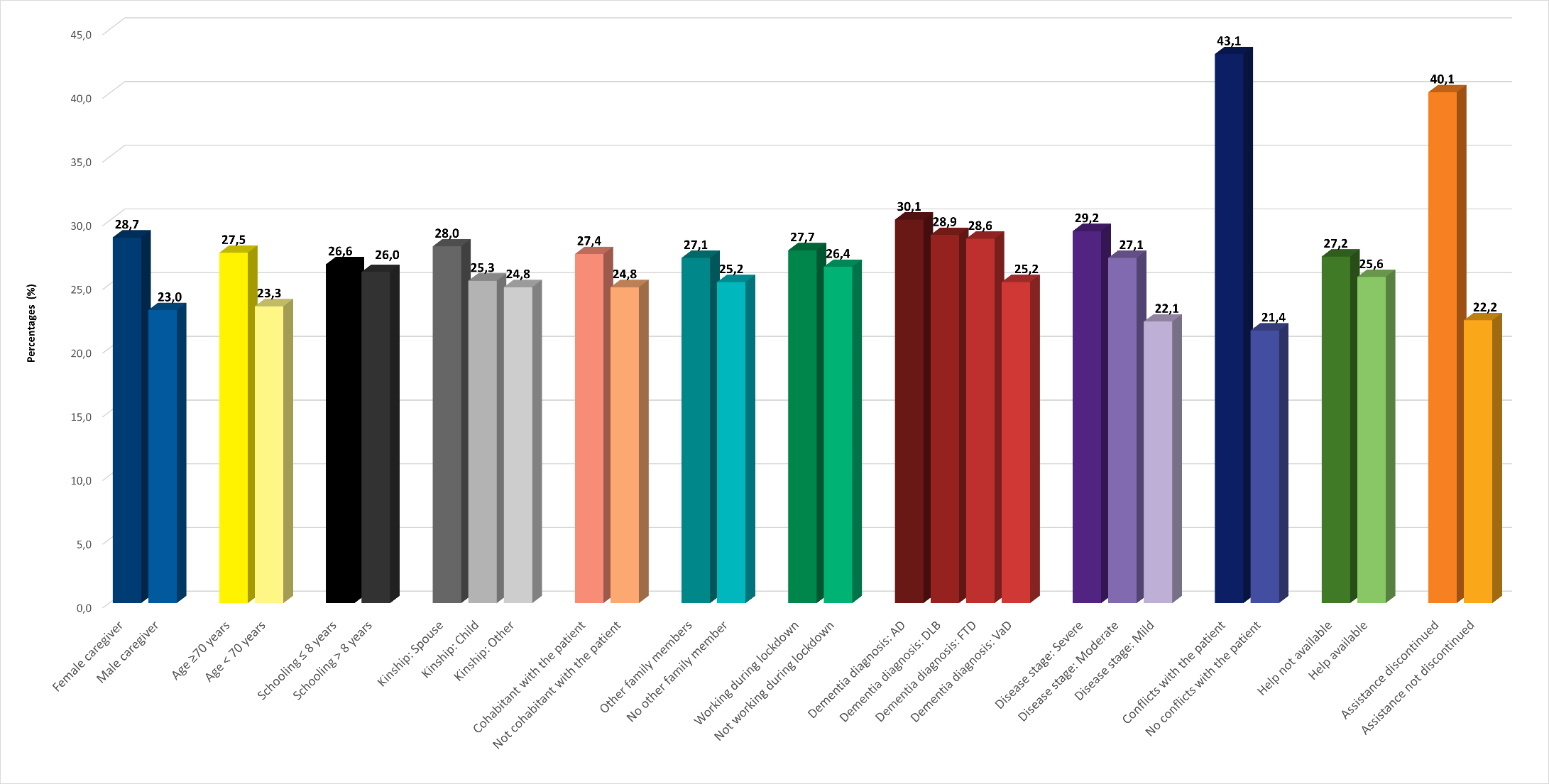


Legend: AD= Alzheimer's Disease, FTD= Frontotemporal Dementia, DLB= Dementia with Lewy bodies, VaD= Vascular Dementia
